# Cryogenian magmatic activity and early life evolution

**DOI:** 10.1038/s41598-019-43177-8

**Published:** 2019-04-29

**Authors:** Jie Long, Shixi Zhang, Kunli Luo

**Affiliations:** 1 Institute of Geographic Sciences and Natural Resources Research, Chinese Academy of Sciences, Beijing, 100101 China; 2 University of Chinese Academy of Sciences, Beijing, 100049 China

**Keywords:** Origin of life, Palaeontology, Palaeontology

## Abstract

Data from the Qinling Orogenic Belt in China indicate that a strong magmatic-volcanic event on the Snowball Earth during the Cryogenian age (approximately 720–635 million years ago) was followed by a dynamic period of accelerated evolution of early life through the Ediacaran period. The studied volcanics of the Cryogenian Yaolinghe group are mainly represented by andesite, dacite and rhyolite, with minor amounts of basalt, trachy andesite and trachyte towards the top, which formed in the environment of an active island arc related to a continental margin. Compared with average felsic volcanics, the studied Cryogenian marine volcanic strata are enriched (1.5–30.6 times) in Co, Cr, Bi, Ni, Se, Ga, As, Cu, Ba, V, and Zn. Elemental concentrations (P, Cd, Co, Ni, and Se) of the studied volcanics are more than 5–26.4 times those in the contemporaneous Liantuo tillite. We propose that Cryogenian magmatic and volcanic activity increased the flux of some trace nutritional elements into the oceans which possibly provided essential nutrients for the development of early life.

## Introduction

The Cryogenian period (ca. 720–635 million years ago) was a period of dynamic environmental change that witnessed the low-latitude glaciation and super continental breakup of Rodina and associated rift-magmatic activity^1,2^. The Sturtian glaciations (ca. 715–680 million years ago) and Marinoan glaciations (ca. 650–635 million years ago) were the most extensive ice times known to have existed on Earth, and they even extended to the equatorial zone and are thus known as “Snowball Earth events”^1,2^. During these periods, glacial sediments were distributed over continents including South China^3,4^, Northern Norway, Eastern Virginia, East Greenland, Scotland, Svalbard, France, Central Africa, Brazil, and Western Australia^5^. These places were positioned at low to intermediate latitudes during the Cryogenian time. At the same times, a large amount of relatively thick (>2000 meters) Cryogenian volcanic-derived sediments and volcanics were widely distributed in these same districts^6–17^. In addition, the Cryogenian played a key role in the evolutionary and developmental history of early life as a link between the preceding and following periods^18,19^. Before the Cryogenian period, life was simple organisms, but afterward, evolutionary processes were characterized by a progressively accelerated stage, with life becoming more complex from 635 to 520 million years ago^20–22^.

**Figure 1:**
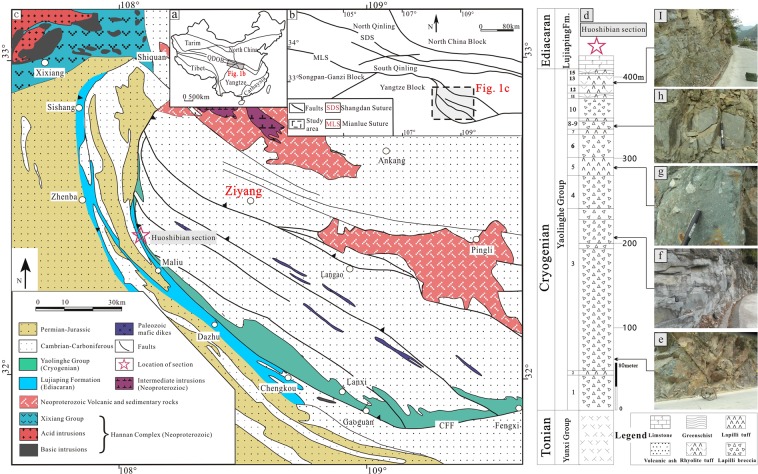
(**a**) Chinese tectonic framework, modified from ref.^23^. (**b**) Sketch map of the Qinling orogenic belt and the relationship between the North China, Qinling, and Yangtze Block domains. (**c**) Brevitied geological map of the southern - South Qingling Orogenic Belt, modified from the ref.^17,26^. CFF: Chengkou-Fengxi Fault. (**d**) Stratigraphic column of the Cryogenian Yaolinghe Group from the Huoshibian Section, at the Maoba town, Ziyang County, Shaanxi Province, Center China. (**e**–**i)** Field occurrence of the Yaolinghe Group volcanic rocks.

The Qinling Orogenic Belt (QOB) is in a significant tectonic position linking the Yangtze and North China Plate (Fig. 1a,b)^23–25^. Here the QOB is divided into the South and North Qinling Orogenic Belt. In the South Qinling Orogenic Belt (SQOB), a large amount of relatively thick Cryogenian marine volcanics and associated sediment had a widespread distribution^6,25,26^, whereas the current Yangtze Plate (South China) adjacent to the southern SQOB contains thick Cryogenian glacial sediments^27,28^. Paleontologists interested in studying the stratigraphy and sedimentology of the Cryogenian glaciation sediment have suggested that the Sturtian and Marinoan glaciations had an important effect on early biological evolution^28–30^, but geologists have paid more attention to the isotopes and geochronology of the Cryogenian volcanic sediment^24,25,31^, providing a new window into tectonic evolution (e.g., the subduction of the oceanic lithosphere and the closure of ocean basins). However, research into the relationship between the Cryogenian magmatic activity and early biological evolution has been uncommon.

Changes in the element abundances of geological time-scale strata are intimately linked to the evolution of life and important environmental changes on Earth^32,33^. The contents of trace elements in organisms show a consistent trend with their abundance in the crust of the earth^34^, which indicates a close relationship between life and natural chemical environments. A number of trace elements are known to have important biological functions that, although present at low levels in organisms^35^, play physiologically significant roles in basic activity functions^36^. It has been suggested that some trace nutrient elements might have contributed to biological evolution throughout geological history^32,37^, but accept for here papers^21,32,37^, there have been few reports on the trace element compositions in whole stratigraphic sequences of Cryogenian marine volcanic strata as well as little research into the possible relationship between these elements and early biological evolution during this period.

A total of 254 volcanic rock samples of the Cryogenian Yaolinghe group (CYG) in 410 meters thickness stratum were collected from the town of Maoba in the Ziyang area of the SQOB in Central China (32°18.188′N-108°13.415′E) (Fig. 1c,d and Table S1). The intervals for these collected rock samples varied from approximately 0.1 to 7 meter, which meant that these collected samples could represent the entire features of the studied Cryogenian strata. In this paper, we report the variations of trace elements of marine volcanic sediments from the Cryogenian strata and their possible correlations with the evolution of early life.

## Lithological Composition and Tectonic Affinity

**Figure 2:**
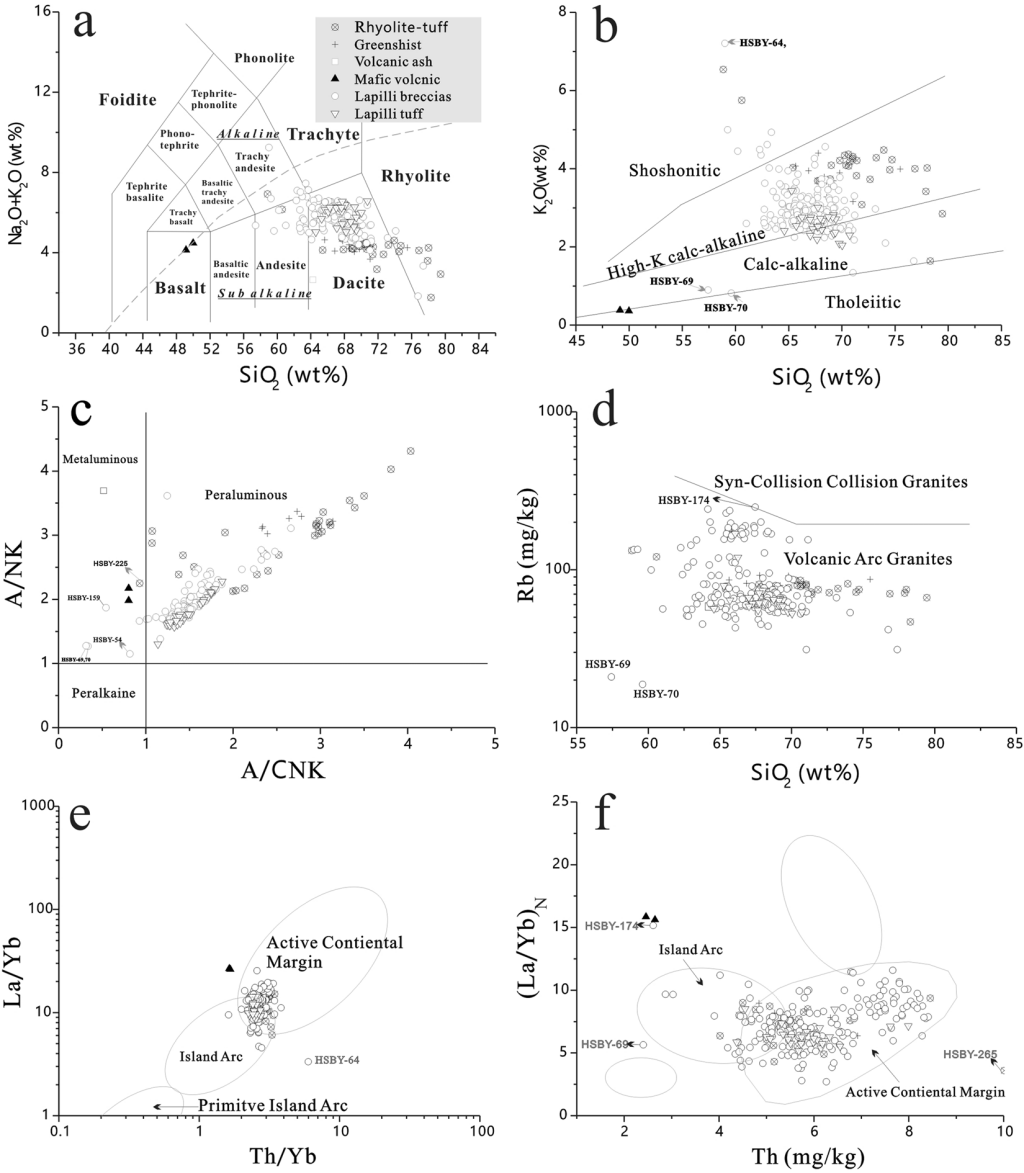
(**a**) Total alkali-silica graph (modified from the ref.^38^) of the Yaolinghe Group from south belt (Ziyang area) of S-SQOB. (**b**) Plot of K_2_O vs. SiO_2_ graph^64^ showing geochemical classification of the Yaolinghe Group from south belt (Ziyang area) of S-SQOB. (**c**) A/CNK vs. A/NK diagram^65^. (**d**) SiO_2_ vs. Rb diagram. (**e**,**f**) w(Th) vs. w(La)/w(Yb) and Th/Yb vs. La/Yb diagram^66^.

The Cryogenian Yaolinghe Group (CYG) is widely distributed in S-SQOB^25,31^, including the north (Yuxi area), middle (An’kang-Pingli area) and south belts (Ziyang area, this study) (Fig. 1c). The studied CYG of Ziyang area (belongs to south belts) show relatively high SiO_2_ (49.14–79.45 wt%, avg. = 67.39 wt%), K_2_O (0.36–7.21 wt%, avg. = 3.06 wt%) and total alkali (ALK = Na_2_O + K_2_O) and low Na_2_O (0.08–4.46 wt%, avg. = 2.46 wt%) (Table S2). According to the total alkali-silica (TAS) classification^38^, the studied CYG is mainly represented by andesite, dacite and rhyolite with minor amounts of basalt, trachy andesite and trachyte (Fig. 2a), and it is characterized by the high-potassium calc-alkaline and calc-alkaline series and is predominantly peraluminous (Fig. 2b,c). As shown in Fig. 2d–f, those volcanics are classified as an island arc related to the active continental margin.

## Elemental Enrichment Patterns

To evaluate the elemental enrichment patterns, concentration coefficients (*CC*s) of the elements in volcanics can be classified into four enrichment patterns (super, anomalous, significant, and slight enrichment), one normal pattern, and one depletion with their corresponding *CC*s of >100, 100 > *CC* > 10, 10 > *CC* > 5, 5 > *CC* > 1.5, 1.5 > *CC* > 0.5, and 0.5 > *CC*. In addition, phosphorus is a very important nutrient^32^ and phosphorus levels are considered as a limiting nutrient in ancient oceans^39^. Thus, phosphorus (P) and other trace elements (including Se, V, Mo, Zn, Cu, Cr, As, Ba, Co, Ni, Sr, Cd, U, Sc, Th, Tl, Rb, Bi, Be, In, Ga and Sc) are selected in this paper for comparison with the average contents of (***1***) the upper continental crust (UCC)^40^, (***2***) Tonian and Cryogenian volcanic rocks from Brazil and India, (***3***) similar lithologies such as intermediate and felsic volcanic rocks^41^, and (***4***) volcanics^6^ and tillites^42^ of nearby areas.

**Figure 3:**
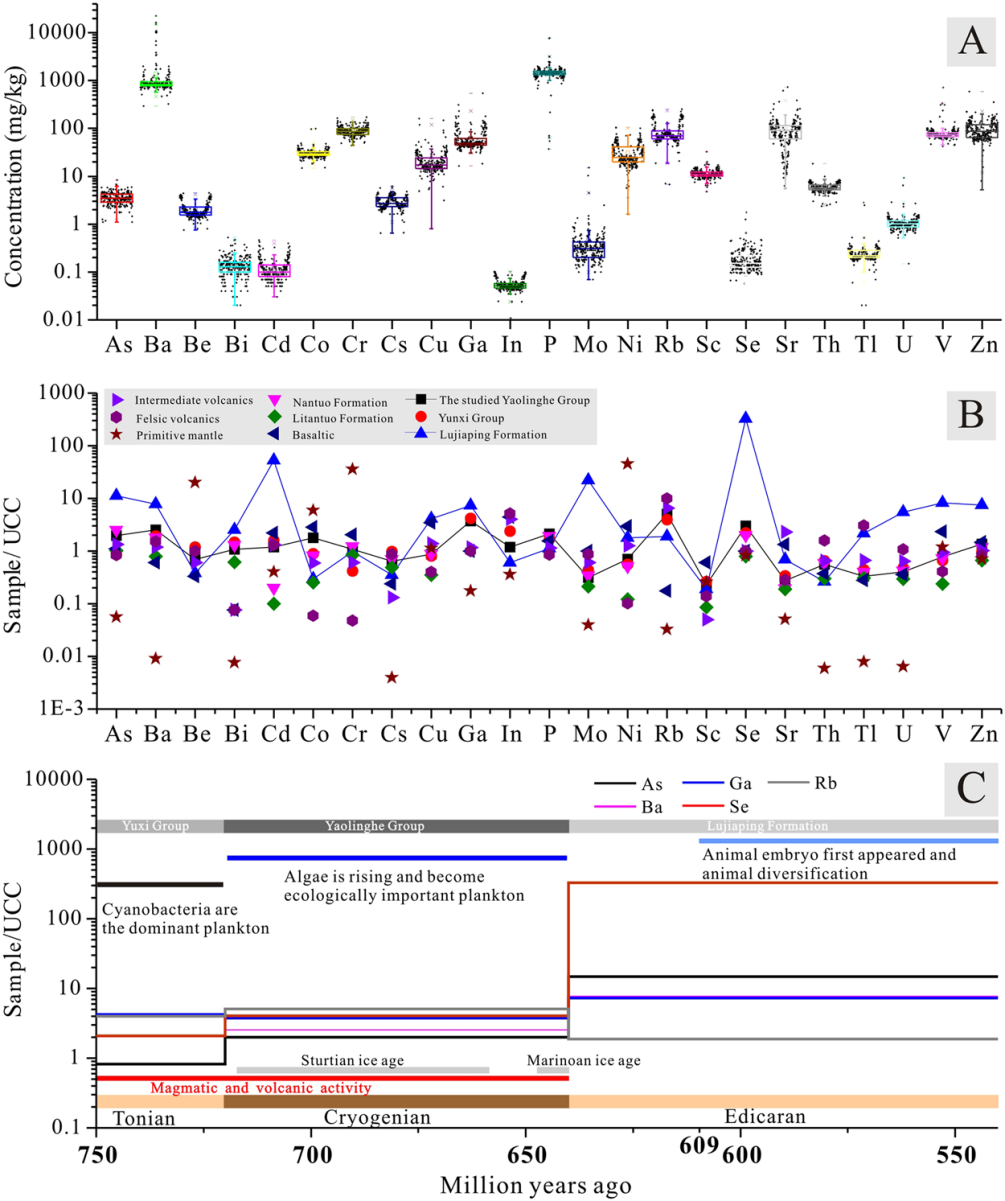
(**a**) The Concentration of the trace elements and phosphorus (P) investigated in the entire studied Cryogenian Yaolinghe strata. (**b**) The CC values of the trace elements from the investigated Cryogenian Yaolinghe strata and its overlain (Lujiaping Formation), covered strata (Yunxi Group), as well as Cryogenian Lantuo and Liantuo tillites strata of nearby areas. (**c**) The average CC of As, Ga, Rb, Ba and Se of Tonian Yunxi group, Cryogenian Yaolinghe group and Edicaran - Lower Cambrian Lujiaping formation, with biological changes (modified from ref.^20^) of that time.

Compared to average element contents in the felsic volcanic rocks^41^, Co, Cr, and Bi are abnormally enriched in the studied CYG with CCs of 30.6, 22.2 and 14, respectively, and Ni is significantly enriched (CC = 7.0). Elements including Se (×4), Ga (×3.7), P (×2.5), As (×2.4), Cu (×2.2), V (×1.9), Ba (×1.7), Sc (×1.6) and Zn (×1.6) are slightly enriched (1.5 < CC < 5), and the contents of the remaining elements, including Sr, Cd, Cs, Be, Rb, U, Mo, Th, In, and Tl, are normal or depleted (Fig. 3a). In addition, compared to globally averaged concentrations of elements in intermediate volcanic rocks, Bi is highly enriched in the studied CYG volcanics with a CC of 14 followed by Cs (×4.9), Sc (×4.6), Se (×4.0) Ga (×3.2), Co (×3.1), Ba (×2.1), Cr (×1.8), and As (×1.5), while the remaining elements are normal or depleted (Fig. 3b and Table S2).

Compared to the contemporaneous volcanics, **(1)** the Cr, Ni, Ga, Cs, Cu, Ba, Rb, Zn, and U values of the studied volcanic samples are 6.3, 3.3, 3.3, 2.5, 2.3, 2.1, 0.7, 1.6, and 1.6 times greater than those of the Wudumeng group volcanics, South China^6^, while the Sc, V, Th, P, Co, and Sr values are lower. **(2)** The *CC*s of Ni, Co, Zn, P, Cu and Ba in the studied samples are 4.8, 3.8, 2.1, 1.5, 1.5 and 1.5 times greater than those of Iriri group volcanics from Northeast Mato Grosso (Brazil)^43^, while those of V, Sc, Cs, Rb, Mo, Th, U, and Sr are close to or lower. **(3)** The Co, Cr, Ni, V, Ga, Ba and Sc values of these samples are 6.1, 4.9, 3.2, 2.5, 2.1, 2.0, and 1.5 times greater than those of Malani group volcanics from the Kundal area (India)^44^, while those of Sr, Zn, Cs, P, Rb, Cu, Th, and U are close to or lower (Fig. 3b and Table S2).

Compared with the Cryogenian tillite from the Yangtze Block (adjacent to the SQOB) in South China^42^, the enrichment pattern of trace elements in marine Yaolinghe volcanic from the SQOB (Central China) are as follows (Fig. 3b and Table S2): (1) P > Cd > Co > Ni > Se > V > Ba > Sc > Cu > Zn > As > Th > Bi > 1.5 > Mo > Sr > U > Cs > Cr > Tl. The average concentrations of P, Cd, Co, Ni, and Se are more than 26.4, 12.0, 7.1, 5.9, and 5.0 times those in Cryogenian Liantuo tillite. (2) Cd > Co > Se > Sr > 1.5 > Ni > Ba > Zn > P > V > Cu > Th > U > Mo > Cs > Cr > Tl > Bi > As. The average concentrations of Cd, Co, Se, and Sr are more than 1.5–6.1 times those in Cryogenian Nantuo tillite.

Compared with the UCC^40^, the CCs of 22 analyzed trace elements within the entire CYG are, from highest to lowest, as follows: Rb > Se > Ga > Ba > P > As > Co > Zn > Cd > Bi > In > Cr > Cu > V > Ni > Be > Cs > Th > U > Tl > Mo > Sr > Sc. Rb is most enriched, and its CCs vary from 0.40 (HSBY 229, mafic volcanic) to 14.7 (HSBY 19, lapilli-breccia) with an average CC value of 5.1 followed by Se × 3.9 (1.2–34.9), Ga × 3.7 (0.9–32.1), Ba × 2.5 (0.5–41), P × 2.13 (10^−3^–11), As × 2.0 (0.6–4.6) and Co × 1.8 (0.9–95.8). The concentrations of the remaining elements are close to or below that in the UCC (Fig. 3b and Table S2).

**Figure 4:**
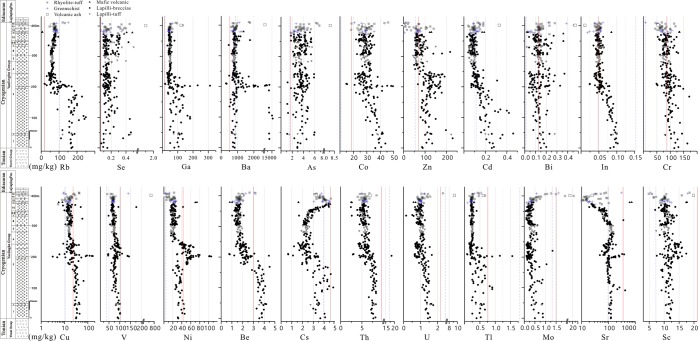
Concentration and distribution patterns of element from the studied Cryogenian Yaolinghe group in ascending order. Red line represent the average concentration of the corresponding element in the UCC (data from ref.^40^), and blue dashed line represent the average concentration of the corresponding element in the felsic volcanics (data from ref.^41^).

Within the entire studied CYG from the highest to lowest parts, the concentrations of Se, As, Ba, Mo, Cs and Sc show increasing trends while those of Rb, Ga, Co, Zn, Cd, Bi, In, Cr, Cu, V, Ni, Be, U, Tl and Sr trend downward (Fig. 4). The contents of Se, As, Zn, V and Ba in the marine rocks from the Tonian Yunxi group (ca. 750 to 720 Ma), the Cryogenian Yaolinghe group (ca. 720 to 635 Ma) and the Ediacaran Lujiaping Formation (ca. 635 to 520 Ma) show increasing trends (Fig. 3c and Table S2).

## Biological Significance of Trace Elements

Rb, Se, Ga, and As are essential microelements in the living eukaryotes, including mammals and humans^35^, that are only present at low levels but are essential to metabolism or for the manufacture of essential biomolecules such as enzymes. Rb^+^ can replace K^+^ as a necessary nutrient for the growth of certain organisms^45,46^, e.g., *Characeae* and *Saccharomyce*, and increasing Rb, Se, Ga and As by various amounts has been proven to promote cell growth^47–50^. The soluble compounds of Ba have been regarded as highly toxic to organisms, but an experiment by Rygh showed that the lack of Ba in feed can degrade the development and calcification of rat and pig (*Cavia porcellus*) bones, while feed containing Ba can promote their development and growth^46^. However, the threshold values of these microelements are generally narrow, and both their deficient and excessive intake influence the survival and development of organisms^51^. Besides, phosphorus (P) is a very important nutrient in the oceans through geological times^32^ and its concentrations could represent the levels of primary productivity in ancient oceans^39^.

## Magmatic Activity and Biological Changes in the Cryogenian Periods

A large amount of Cryogenian intermediate felsic volcanics occur widely in the Southern-SQOB, while Cryogenian basaltic volcanics are also distributed throughout the Middle and Northern-SQOB; the entire Southern-SQOB was located in the Paleo Asian Ocean during that time^52^. In addition, a large amount of very thick (>2000 meters) Cryogenian volcanic sediments were not only present in the Qinling-Dabie-Sulu orogen of Central China^6,7^ but also widespread on most continents, including the Seychelles in the Indian Ocean^8,9^, Madagascar of Africa^10,11^, India^12,13^, Canada^14^ and Australia^15,16^. This suggests there were large-scale and sustained magmatic and volcanic activities in the Paleo Asian Ocean in the period of ca. 720–635 Ma and that such activities in SQOB (Central China) were not just local; they could have been an integral part of global magmatism and volcanism during the Cryogenian period.

In modern oceans, many new species exist and develop in deep-sea hydrothermal vent environments such as black smoker vent water (~300 °C/1369 meters), black smoker chimneys (~300 °C/1369 meters) and water-simmering sediments (~100 °C/1398 meters)^53–55^, these environments are characterized by high-temperature fluids containing relatively high concentrations of Sr, Ba, S, Ca, and transition metals deposited from carbonate-rich hydrothermal precipitates with disseminated sulfides^54^. In the hydrothermal vent sites of ancient oceans (ca. 520 million years ago), few submarine animals and abundant algae (*Cyanophytaor Chlorophyta*) fossils have been observed in barite deposits (South China) developed at 180 °C^56^. Few worm fossils and abundant fossils of large sponges, bivalves, and algae were found at early Cambrian Mo-Ni sulfide black shales (Zunyi County, Guizhou Province, South China)^57,58^, and worm fossils were discovered in Cretaceous sulfide black shale (Samal Ophiolite, Oman)^59^.

During the Tonian period (ca. 750–720 Ma), Cyanobacteria were the dominant plankton^20^, but over the next 85 million years, algae evolved and became ecologically important^20^. Animal embryos first appeared ca. 609 Ma^60,61^, and animals began to diversify during the Ediacaran time^22,62^ (Fig. 3c). The variation and diversification of these organisms from ca. 750 to 541 Ma^20^ may have corresponded to the trend of increasing Se, As and Ba contents in rocks from the Neoproterozoic Era (Fig. 3c). This is supported from the clear evidence of nutrient changes (including Se, Cd, Co, and P) changes in the pyrite from 700 million years ago to the Cambrian explosion^26^. Average concentrations of P, Cd, Co, Ni, and Se from the studied volcanics are more than 26.4, 12.0, 7.4, 5.9, and 5.0 times those in the contemporaneous Liantuo tillite (Table S2). Additionally, Rb is enriched in Cryogenian Malani group volcanics (Kundal area, India) and Iriri group volcanics (Northeast Mato Grosso, Brazil), and its average concentration is 150.9 and 289.3 mg/kg, which is 8.9 and 17.0 times greater than those of the UCC (17 mg/kg)^40^. Se in the studied samples varies from 0.06 to 1.7 mg/kg, with a mean of 0.2 mg/kg, which is 2.0, 5.0, 4.0 and 4.0 times more than that in the contemporaneous Niantuo tillite, Liantuo tillite, the UCC and felsic volcanics, respectively. In addition, relative high Se and As concentration was found at the Cryogenian marine pyrite, with an arithmetic mean of 48.5 and 564.1 mg/kg^37^, which is 969 and 313 times higher than those of the UCC.

Thus, the above results provide new clues that although the global surface was overlain by very thick ice during the period of Cryogenian glaciations, a global magmatic and volcanic event occurred that not only guaranteed longtime-favorable and warm-water conditions for the survival of certain oceanic organisms but also carried an essential concentration of nutrient trace elements (such as slight enrichment of Se and P) to the oceans. We propose that Cryogenian magmatic and volcanic activity increased the flux of some trace nutritional elements into the oceans which possibly provided essential nutrients for the development of early life.

## Samples and Methods

### Sample collection

Cryogenian marine volcanic-sedimentary sequences of the Yaolinghe Group cover an area of ~6100 Km2 from Zhenba in the west to Xichuan in the east and have an average thickness of more than 1000 m (300–3600 meters) in the South Qinling Orogenic Belt (SQOB) (Fig. 1c,d)^7^. The Yaolinghe Group of the studied area consists of intermediate-acid volcanic rocks, and this Group is divided into 15 units in ascending order, on the basis of occurrence in the field, lithological characteristics and major element compositions (Fig. 1c,d and Table S1). The Yaolinghe Group yields U-Pb ages ranging from 720 million years ago (Ma) to 632 ± 1 Ma^31^.

During Sep. 2014, a total of 735 fresh rock samples were collected at approximately 1000 meters from units in the Cryogenian Yaolinghe group and the Ediacaran Lujiaping formation at the Huoshibian Section of the town of Maoba in Ziyang County (Shaanxi Province, China) and the data of 466 rock samples from the Ediacaran- Lower Cambrian Lujiaping formation were cited from the Long and Luo (2016)^63^. The locations and thicknesses of the 254 fresh volcanic rock samples from the Cryogenian Yaolinghe group were shown in Table S1 and Fig. 1d.

### Sample analysis

All volcanic samples of the Yaolinghe group were groud and analyzed at the LATC (Laboratory Analysis and Testing Center), IGSNRR (Institute of Geographic Sciences and Natural Resources Research), CAS (Chinese Academy of Sciences), Beijing, China. The detailed process for sample dissolution and calibration has been reported and noted by Luo (2011), Ni (2016), and Long and Luo (2016)^63^, and the GBW07107 and GBW07112 China rock standards were used in the analysis as well as two groups of parallel samples. At the same time, repeated measurements and a blank experiment were also conducted, which ensured its precision and accuracy of the datas. The concentrations of elements were tested by ICP-OES and ICP-MS, and Se and As were tested by an HG-AFS manufactured by Beijing Haiguang Instruments Co. Ltd.

## Supplementary information

Supplementary tables
