# Supplementary material for: Cryogenian magmatic activity and early life evolution
Source: Sci Rep. 2019 Apr 29;9:6586. doi: 10.1038/s41598-019-43177-8 (PMC6488696; doi:10.1038/s41598-019-43177-8)
Supplement: Supplementary file 1 — Supplementary tables [file 41598_2019_43177_MOESM1_ESM.doc]

Supplementary Information for

**Cryogenian magmatic activity and early life evolution**

Jie Long^1,2^*, Shixi Zhang^1^*, Kunli Luo1,2†[[1]](#footnote-2)

Correspondence author：Kunli Luo

Email: luokl@igsnrr.ac.cn

This file includes:

Table S1, and S2

.

**Table S1 The detailed description of 15 units of the Cryogenian Yaolinghe group from Southern-** **South Qinling Orogenic Belt, China**.

Abbreviation: F.=Formation. T.=Thickness.

| **F.** | **Unit** | **T.** | **Sample Number.** | **Lithology ant its description** | |
| --- | --- | --- | --- | --- | --- |
| Ediacaran Lujiaping formation | | | | Dark-black siliceous, shale, dolomite and interbedded with minor, barite ore and volcanic tuff | |
| Cryogenian Yaolinghe Group | unit 15 | 4m | n=6, HSBY259 to 265 | Gray green colored rhyolite-tuff and greenschist. | |
|  | unit 14 | 0.1m | n=1, HSBY258 | Gray-white colored volcanic ash. | |
|  | unit 13 | 12m | n=20, HSBY236 to 257 | Gray-white or gray-cyan colored rhyolite-tuff. | |
|  | unit 12 | 5m | n=8, HSBY227 to 235 | Khaki or gray-yellow colored and banded rhyolite-tuff, interbedded with mafic volcanics (ca. 0.9 m-thick). Fine grained (maximum grain size less than 1mm). Sometimes pyrite are also present. | |
|  | unit 11 | 4m | n=6 HSBY220 to 225 | Cyan or gray-green colored rhyolite-tuff and greenschist. | |
|  | unit 10 | 28m | n=34, HSBY186 to 219 | Gray or gray-green colored lapilli-breccia. Massive and breccia structure. Fine grained | |
|  | unit 9 | 1.8m | n=4, HSBY182 to 185 | Gray-yellow colored lapilli-tuff. Massive structure. Poor-sorted and white medium grained. | |
|  | unit 8 | 4.1m | n=11, HSBY171 to 181 | Light lime-green or gray-cyan colored lapilli-breccia. Massive and breccia structure. White medium grained, Clasts (no directionality) are subangular to subrounded. Rounded clasts dominate. | |
|  | unit 7 | 3.8m | n=6, HSBY165 to 170 | Khaki or gray-yellow colored lapilli-tuff, with massive structure. Poor-sorted. | |
|  | unit 6 | 32m | n=29, HSBY136 to 164 | Light lime-green colored lapilli-breccia. Medium to coarse grained. Clasts (no directionality) are subangular to subrounded, sometimes sharp corners. | |
|  | unit 5 | 19m | n=14, HSBY122 to 135 | Gray or gray-yellow colored lapilli-tuff, with massive structure and fine grained. | |
|  | unit 4 | 36m | n=16, HSBY106 to 121 | Gray-cyan or gray-green colored lapilli-breccia, with massive structure and fine grained. | |
|  | unit 3 | 15m | n=14, HSBY89 to 105 | Gray-green or dark gray-green colored lapilli-breccia. | Massive and breccias structure; Poor-sorted, large proportion of clasts;Clasts(no directionality) are subangular to subrounded, sometimes sharp corners; high proportion of siliciclastic detritus; Sometimes pyrite are also present. |
|  |  | 29.4m | n=26, HSBY63 to 88 | Dark gray or gray-green colored lapilli-breccia. White coarse grained. |  |
|  |  | 5.2m | n=8, HSBY53 to 62 | Brown or reddish colored lapilli-breccia; white and gray coarse grained. Triangular and quadrangular columnar jointing. |  |
|  |  | 142m | n=42, HSBY10 to 52 | Gray green colored lapilli-breccia. white and black coarse grained, sometimes reddish colored. |  |
|  | unit 2 | 3.1m | n=2, HSBY-8 to 9 | Khaki or gray-yellow colored lapilli-tuff. Massive structure. Poor-sorted and medium grained clasts with sharp corners. | |
|  | unit 1 | 43m | n=7, HSBY-1 to 7 | Dark green colored lapilli-breccia. Massive and lapilli-breccia structure. Poor-sorted. Grey coarse grained matrix and variable content of macroscopically visible clasts. Clasts (no directionality) are subangular to subrounded. Cross-bedding. | |
| Tonian Yunxi group | | | | Intermediate-acid volcanic rocks[^3^](#_ENREF_3) | |

**Table S2 Trace elements of the Cryogenian Yaolinghe group volcanics of Southern-SQOB, China, and Cryogenian volcanics and tillites from other area (mg/kg)**

|  | China | | | | | | | | | | Brazil | Indian | UCC | Basaltic | In. V | Fe. V | Pm |
| --- | --- | --- | --- | --- | --- | --- | --- | --- | --- | --- | --- | --- | --- | --- | --- | --- | --- |
|  | South Qingling Orogenic Belt | | | | | | | South China Block | | | Ir. V | Ml. V |  |  |  |  |  |
|  | The Yaolinghe Group volcanics | | | | | YXg | LFm | Wd. V | Nt. T | Lt.T |  |  |  |  |  |  |  |
|  | This study | | | An'kan | Yunxi |  |  |  |  |  |  |  |  |  |  |  |  |
|  | Min. | Max. | Avg. |  |  |  |  |  |  |  |  |  |  |  |  |  |  |
| As | 1.10 | 8.30 | 3.57 | - | - | 1.55 | 20.12 | - | 4.59 | 1.85 | - | - | 1.80 | 2.00 | 2.40 | 1.50 | 0.10 |
| Ba | 294 | 22530 | 1392 | 380 | 145 | 1056 | 4295 | 646 | 1000 | 440 | 1213 | 697 | 550 | 330 | 650 | 840 | 5 |
| Be | 0.75 | 4.51 | 2.06 | - | - | 3.58 | 1.13 |  | 0.00 | 0.00 | - | - | 3.00 | 1.00 | 1.80 | 3.00 | 60 |
| Bi | 0.02 | 0.53 | 0.14 | - | - | 0.19 | 0.33 |  | 0.17 | 0.08 | - | - | 0.13 | 0.01 | 0.01 | 0.01 | 0.001 |
| Cd | 0.03 | 0.46 | 0.12 | - | - | 0.16 | 5.24 |  | 0.02 | 0.01 | - | - | 0.10 | 0.22 | 0.13 | 0.13 | 0.040 |
| Co | 14.73 | 99.17 | 30.62 | 15.96 | 42.19 | 15.19 | 5.05 | 84.33 | 13.99 | 4.33 | 8.16 | 5.03 | 17.00 | 48.00 | 10.00 | 1.00 | 100 |
| Cr | 43.85 | 170.80 | 88.90 | 71.73 | 200.30 | 34.90 | 80.84 | 14.03 | 103.55 | 69.65 | - | 17.94 | 83.00 | 170.00 | 50.00 | 4.00 | 3000 |
| Cs | 0.65 | 6.25 | 2.94 | 1.38 | 1.63 | 4.48 | 1.61 | 1.17 | 3.27 | 2.26 | 4.88 | 8.22 | 4.60 | 1.10 | 0.60 | 4.00 | 0.018 |
| Cu | 0.00 | 161.80 | 21.93 | - | - | 20.36 | 102.20 | 9.51 | 22.35 | 8.87 | 14.46 | 52.99 | 25.00 | 87.00 | 35.00 | 10.00 | 28 |
| Ga | 16.49 | 546.16 | 63.66 | 22.00 | 13.50 | 70.73 | 125.12 | 19.29 | - | - | - | 30.92 | 17.00 | 17.00 | 20.00 | 17.00 | 3 |
| In | 0.01 | 0.10 | 0.06 | - | - | 0.12 | 0.03 |  | - | - | - | - | 0.05 | 0.22 | 0.20 | 0.26 | 0.018 |
| P | 3.7 | 7720 | 1490 | 1228 | 3233 | 1184 | 1602 | 1790 | 1348 | 56.52 | 981 | 1562 | 700 | 1100 | 800 | 600 | - |
| Mo | 0.07 | 15.14 | 0.47 | - | - | 0.65 | 32.87 |  | 0.52 | 0.32 | 0.85 | - | 1.50 | 1.50 | 0.90 | 1.30 | 0.059 |
| Ni | 0.00 | 105.50 | 31.30 | 39.01 | 91.37 | 24.47 | 77.98 | 9.37 | 22.86 | 5.35 | 6.49 | 9.85 | 44.00 | 130.00 | 55.00 | 4.50 | 2000 |
| Rb | 7.00 | 249.57 | 86.00 | 33.11 | 13.02 | 67.58 | 32.02 | 49.75 | - | - | 150.88 | 289.28 | 17.00 | 3.00 | 110.00 | 170.00 | 0.55 |
| Sc | 4.85 | 32.82 | 11.46 | 21.33 | 32.64 | 13.21 | 9.51 | 8.90 | - | 4.29 | 10.00 | 7.69 | 50.00 | 30.00 | 2.50 | 7.00 | 13 |
| Se | 0.06 | 1.75 | 0.20 | - | - | 0.11 | 16.40 | - | 0.10 | 0.04 | - | - | 0.05 | 0.05 | 0.05 | 0.05 | 0.041 |
| Sr | 6.00 | 738.30 | 95.34 | 246.44 | 220.77 | 117.76 | 243.07 | 575.15 | 69.03 | 66.30 | 476.61 | 85.21 | 350.00 | 465.00 | 800.00 | 100.00 | 17.8 |
| Th | 2.40 | 18.34 | 5.97 | 6.17 | 2.34 | 7.05 | 2.82 | 5.66 | 6.34 | 3.24 | 14.38 | 44.11 | 10.70 | 4.00 | 7.00 | 17.00 | 0.064 |
| Tl | 0.02 | 2.83 | 0.25 | - | - | 0.33 | 1.64 | - | 0.30 | 0.23 | - | - | 0.75 | 0.21 | 0.50 | 2.30 | 0.006 |
| U | 0.15 | 9.30 | 1.12 | 1.45 | 0.75 | 1.34 | 15.39 | 0.72 | 1.19 | 0.83 | 3.64 | 8.53 | 2.80 | 1.00 | 1.80 | 3.00 | 0.018 |
| V | 42.00 | 715.50 | 83.39 | 124.03 | 279.73 | 71.53 | 879.38 | 65.15 | 78.07 | 25.47 | 60.36 | 32.92 | 107.00 | 250.00 | 100.00 | 44.00 | 128 |
| Zn | 0.00 | 592.90 | 96.09 | - | - | 69.74 | 526.07 | 61.38 | 85.26 | 47.45 | 44.93 | 235.73 | 71.00 | 105.00 | 72.00 | 60.00 | 50 |

**The Yaolinghe Group is widely distributed in S-SQOB, including north (Yuxi area), middle (An’kang area) and south belt (Ziyang area, this sduty). Abbreviation:** Min.=Minium. Max.=Maxium. Avg.=Average. YXg= Yunxi Group volcanics (ca. 750 Ma~720), data from [^3^](#_ENREF_3). LFm=Lujiaping Formation (ca. 635 Ma~541) data from [^2^](#_ENREF_2). Wd.V= Wudumeng Formation volcanics (ca. 730 Ma~), data from [^4^](#_ENREF_4). Nt. T=Nantuo formaion tillites (ca. 651~636.3±4.9Ma), data from [^5^](#_ENREF_5). Lt.T=Liantuo formation tillites (ca. 746Ma~?), data from [^5^](#_ENREF_5). Ir. V=Iriri group volcanics (ca. 760~700 Ma), data from [^6^](#_ENREF_6). Ml.V= Malani group volcanics (ca. 725Ma~?), data from [^7^](#_ENREF_7). UCC=Upper Continental Crust, data from [^8^](#_ENREF_8). Bas.=BasalticIn, data from [^9^](#_ENREF_9). V= Intermediate volcanics. Fe. V=Felsic volcanics, data from [^9^](#_ENREF_9). Pm=Primitive mantle, data from [^10^](#_ENREF_10). "-"=No data.

# References

1 Luo, K. L. The lujiaping formtion of northen daba mountain. *Journal of Stratigraphy* **30**, 149-156 (2006).

2 Long, J. & Luo, K. Trace element distribution and enrichment patterns of Ediacaran-early Cambrian, Ziyang selenosis area, Central China: Constraints for the origin of Selenium. *Journal of Geochemical Exploration* **172**, 211-230 (2017).

3 Xia, L. Q., Xia, Z. C., Li, X. M., Ma, Z. P. & Xu, X. Y. Petrogenesis of the Yaolinghe Group, Yunxi Group, Wudangshan Group volcanic rocks and basic dyke swarms from eastern part of the South Qinling Mountains. *Northwestern Geology* **41**, 1-29 (2008).

4 Dong, Y. *et al.* Neoproterozoic accretionary tectonics along the northwestern margin of the Yangtze Block, China: Constraints from zircon U–Pb geochronology and geochemistry. *Precambrian Research* **196-197**, 247-274 (2012).

5 Wang, S. B. The distribution and variation of biological trace elements in Cryogenian of West-northern Hunan Provience, and their biological and environmental response (In Chinese). [Ph.D dissertation.]. *The Institute of Geographic Sciences and Natrual Resources Reserch,Chnese Academy of Sciences, Beijing, China* (2016).

6 Rocha, M. L. B. P., Lima, E. F. D. & Pierosan, R. Paleoproterozoic Domo of Lava from Iriri Group – Sonho Meu Farm – Northeast of Mato Grosso, Amazon Craton: geology, geochemistry, and geochronology. *Revista Brasileira De Geociencias* **42**, 471-488 (2012).

7 Eby, N. & Kochhar, N. Geochemistry and petrogenesis of the Malani igneous suite, North Peninsular India. *Journal of the Geological Society of India* **36**, 109-130 (1990).

8 Taylor, S. R. & Mclennan, S. M. *The Continental Crust: Its Composition and Evolution, An Examination of the Geochemical Record Preserved in Sedimentary Rocks*. (Blackwell Scientific Pub., 1985).

9 Turekian, K. K. & Wedepohl, K. H. Distribution of the Elements in Some Major Units of the Earth's Crust. *Geological Society of America Bulletin* **72**, 175 (1961).

10 Sun, S. S. Chemical composition and origin of the Earth’s primitive mantle.’ Geochim. Cosmochim. Acta 46, 179-192. *Geochimica Et Cosmochimica Acta* **46**, 179-192 (1982).

1. *^1^Institute of Geographic Sciences and Natural Resources Research, Chinese Academy of Sciences, Beijing, 100101.*

   *^2^University of Chinese Academy of Sciences, Beijing, 100049.*

   **These authors contributed equally to this work.*

   *†Correspondence author. Email:* [*luokl@igsnrr.ac.cn*](mailto:luokl@igsnrr.ac.cn) [↑](#footnote-ref-2)
